# Supplementary material for: Mechanosensation of the heart and gut elicits hypometabolism and vigilance in mice
Source: bioRxiv. 2023 Jul 1:2023.06.29.547073. Preprint. [Version 1] doi: 10.1101/2023.06.29.547073 (PMC10327188; doi:10.1101/2023.06.29.547073)
Supplement: Supplement 1 [file media-1.pdf]

Figure	Part		Sample size	Test	P-value, F- value, df, t-values
1	k	IGLEs/mm duodenum	n= 3 males, 3 females	Two-way ANOVA, Šídák's multiple comparisons	Sex F (1,60)= 75.67, P<0.0001; Distance F (14, 60)= 12.37, P<0.0001; Sex x Distance F (14,60)= 8.694, P<0.0001

Figure	Part		Sample size	Test	P-value, F- value, df, t-values
3	b	Food Intake	n= 12 CON-NDG^{Oxtr}, n= 22 Gq-NDG^{Oxtr}	Two-way RM ANOVA followed with Dunnett's multiple comparisons	Time F (19,1134)=364.4, P<0.0001; Treatment F (3,63)=18.20, P<0.0001; Time x Treatment F (54,1134)=7.408, P<0.0001
3	c	VO2	n= 12 CON-NDG^{Oxtr}, n= 22 Gq-NDG^{Oxtr}	Mixed effects analysis with Dunnett's multiple comparisons	Time F (5.510, 331.2)= 25.02, P<0.0001; Treatment F (3,63)=12.72, P<0.0001; Time x Treatment F (54, 1082)= 7.305, P<0.0001
3	d	RER	n= 12 CON-NDG^{Oxtr}, n= 22 Gq-NDG^{Oxtr}	Mixed effects analysis with Dunnett's multiple comparisons	Time F(3.744, 225.7)= 27.31, P<0.0001; Treatment F (3, 64)= 12.79, P<0.0001; Time x Treatment F (54, 1085)= 11.72, P<0.0001
3	e	Temp	n= 4 CON-NDG^{Oxtr}, n= 5 Gq-NDG^{Oxtr}	Two-way RM ANOVA followed with Dunnett's multiple comparisons	Time F(18, 252)= 7.435, P<0.0001; Treatment F (3, 14)= 3.960, P= 0.0309; Time x Treatment F (54, 252)= 8.044, P<0.0001
3	f	Systolic Blood Pressure	n= 4 CON-NDG^{Oxtr}, n= 5 Gq-NDG^{Oxtr}	Two-way RM ANOVA followed with Dunnett's multiple comparisons	Time F(18, 252)= 12.09, P<0.0001; Treatment F (3, 14)= 0.3009, P= 0.8242; Time x Treatment F (54, 252)= 2.896, P<0.0001
3	g	Heart Rate	n= 4 CON-NDG^{Oxtr}, n= 5 Gq-NDG^{Oxtr}	Two-way RM ANOVA, Dunnett's multiple comparisons	Time F(18, 252)= 12.25, P<0.0001; Treatment F (3, 14)= 9.495, P= 0.0011; Time x Treatment F (54, 252)= 6.817, P<0.0001
3	h	Water Intake	n= 12 CON-NDG^{Oxtr}, n= 22 Gq-NDG^{Oxtr}	Two-way RM ANOVA, Dunnett's multiple comparisons	Time F (1.134, 71.42)=345.7, P<0.0001; Treatment F (3,63)=11.70, P<0.0001; Time x Treatment F (54,1134)=5.343, P<0.0001
3	j	total fos AP	n= 4 CON-NDG^{Oxtr}, n= 4 Gq-NDG^{Oxtr}	Unpaired one-way t-test	p= 0.0183, t= 2.677, df= 6
3	j	total fos NTS	n= 4 CON-NDG^{Oxtr}, n= 4 Gq-NDG^{Oxtr}	Unpaired one-way t-test	p= 0.0018, t= 4.618, df= 6
3	j	GIPRr+ Fos+/GIPR+ AP	n= 4 CON-NDG^{Oxtr}, n= 4 Gq-NDG^{Oxtr}	Unpaired one-way t-test	p= 0.0684, t= 1.716, df= 6

Figure	Part		Sample size	Test	P-value, F- value, df, t-values
3	j	GIPR+ Fos+/GIPR+ NTS	n= 4 CON-NDG^{Oxtr}, n= 4 Gq-NDG^{Oxtr}	Unpaired one-way t-test	p= 0.2977, t= 0.5604, df= 6
3	j	VGAT+Fos+/VGAT+ AP	n= 4 CON-NDG^{Oxtr}, n= 4 Gq-NDG^{Oxtr}	Unpaired one-way t-test	p= 0.0257, t= 2.427, df= 6
3	j	VGAT+Fos+/VGAT+ NTS	n= 4 CON-NDG^{Oxtr}, n= 4 Gq-NDG^{Oxtr}	Unpaired one-way t-test	p= 0.0007, t= 5.607, df= 6
3	j	GIPR+Fos+VGAT+/GIPR +VGAT+ AP	n= 4 CON-NDG^{Oxtr}, n= 4 Gq-NDG^{Oxtr}	Unpaired one-way t-test	p= 0.0053, t= 3.651, df= 6
3	j	GIPR+Fos+VGAT+/GIPR +VGAT+ NTS	n= 4 CON-NDG^{Oxtr}, n= 4 Gq-NDG^{Oxtr}	Unpaired one-way t-test	p=0.0087, t= 3.252, df= 6
3	l	total c-Fos PBN	n= 5 CON-NDG^{Oxtr}, n= 5 Gq-NDG^{Oxtr}	Unpaired one-way t-test	p= 0.0479, t= 1.887, df= 8
3	l	CALCA+Fos+/CALCA+ PBN	n= 5 CON-NDG^{Oxtr}, n= 4 Gq-NDG^{Oxtr}	Unpaired one-way t-test	p<0.0001, t= 7.692, df= 7
3	m	Flavor Preference	n= 6 CON-NDG^{Oxtr}, n= 7 Gq-NDG^{Oxtr}	Two-way RM ANOVA, Šídák's multiple comparisons	Injection F(1, 11)= 5.189, P<0.0437; Treatment F (1, 11)= 5.422, P= 0.0400; Time x Treatment F (1, 11)= 17.53, P<0.0015
3	o	Total c-Fos PVN	n= 4 CON-NDG^{Oxtr}, n= 4 Gq-NDG^{Oxtr}	Unpaired one-way t-test	p= 0.0044, t= 3.807, df= 6
3	o	CRH+ c-Fos+/CRH+pvn	n= 4 CON-NDG^{Oxtr}, n= 4 Gq-NDG^{Oxtr}	Unpaired one-way t-test	p= 0.0031, t= 4.111, df= 6
3	p	Plasma Corticosterone	n= 5 CON-NDG^{Oxtr}, n= 5 Gq-NDG^{Oxtr}	Two-way RM ANOVA, Fisher's LSD	Time F(2.184, 17.47)= 16.53, P<0.0001; Treatment F (1, 8)= 18.82, P= 0.0025; Time x Treatment F (4, 32)= 7.927, P= 0.0001

Figure	Part		Sample size	Test	P-value, F- value, df, t-values
4	a	PM Food Intake	n= 13 Gq-NDG^{Oxtr}	Two-way RM ANOVA, Dunnett's multiple comparisons	Time F (21,756)= 5.880, P<0.0001; Treatment F (2, 36)= 10.61, P<0.0001; Time x Treatment F (42,756)= 5.880, P<0.0001
4	b	PM Temp	n= 8 Gq-NDG^{Oxtr}	Two-way RM ANOVA, Dunnett's multiple comparisons	Time F (23, 483)= 11.08 , P<0.0001; Treatment F (2, 21)= 14.32, P=0.0001; Time x Treatment F (46, 483)= 20.61 , P<0.0001
4	c	AM Food Intake	n= 8 Gq-NDG^{Oxtr}	Mixed-effects analysis, Dunnett's multiple comparisons	Time F (1.372, 28.70)= 280.8, P<0.0001; Treatment F (2,21)= 6.808, P<0.0053; Time x Treatment F (46, 481)= 2.388 , P<0.0001
4	d	AM Temp	n= 8 Gq-NDG^{Oxtr}	Two-way RM ANOVA, Dunnett's multiple comparisons	Time F (2.752, 57.79)= 53.18, P<0.0001; Treatment F (2, 21)= 7.793, P=0.0029; Time x Treatment F (46, 483)= 8.032, P<0.0001
4	e	PM VO2	n= 13 Gq-NDG^{Oxtr}	Mixed-effects analysis followed with Dunnett's multiple comparisons	Time F (5.617, 197.1)= 37.57 , P<0.0001; Treatment F (2, 36)= , P= 0.3764; Time x Treatment F (44, 772)= 11.33, P<0.0001
4	f	PM RER	n= 13 Gq-NDG^{Oxtr}	Mixed-effects analysis, Dunnett's multiple comparisons	Time F (21, 732)= 31.52, P<0.0001; Treatment F (2, 36)= 4.393, P=0.196; Time x Treatment F (42,732)= 18.38, P<0.0001
4	g	AM VO2	n= 8 Gq-NDG^{Oxtr}	Mixed-effects analysis, Dunnett's multiple comparisons	Time F (5.776, 120.5)= 49.18 , P<0.0001; Treatment F (2, 21)= 0.4999, P=0.6136; Time x Treatment F (46, 480)= 2.858, P<0.0001
4	h	AM RER	n= 8 Gq-NDG^{Oxtr}	Mixed-effects analysis, Dunnett's multiple comparisons	Time F (3.616, 75.47)= 75.74, P<0.0001; Treatment F (2, 21)= 4.345 , P=0.0264; Time x Treatment F (46, 480)= 3.926, P<0.0001

Figure	Part		Sample size	Test	P-value, F- value, df, t-values
4	i	PM SBP	n= 8 Gq-NDG^{Oxtr}	Two-way RM ANOVA, Dunnett's multiple comparisons	Time F (6.018, 126.4)= 10.23 , P<0.0001; Treatment F (2, 21)= 0.5895 , P=0.5635; Time x Treatment F (46,483)= 4.701, P<0.0001
4	j	PM HR	n= 8 Gq-NDG^{Oxtr}	Two-way RM ANOVA, Dunnett's multiple comparisons	Time F (4.374, 91.85)= 7.973 , P<0.0001; Treatment F (2, 21)= 11.74 , P= 0.0004; Time x Treatment F (46, 483)= 19.88 , P<0.0001
4	k	AM SBP	n= 8 Gq-NDG^{Oxtr}	Two-way RM ANOVA, Dunnett's multiple comparisons	Time F (3.617, 75.95)= 20.34 , P<0.0001; Treatment F (2,21)= 0.3491, P= 0.7094; Time x Treatment F (46, 483)= 1.739, P= 0.0026
4	l	AM HR	n= 8 Gq-NDG^{Oxtr}	Two-way RM ANOVA, Dunnett's multiple comparisons	Time F (6.122, 128.6)= 50.45, P<0.0001; Treatment F (2, 21)= 12.25, P= 0.0003; Time x Treatment F (46, 483)= 8.336, P<0.0001
4	m	PM Body Weight	n= 17 CON-NDG^{Oxtr}, r	Mixed-effects analysis, Šídák's multiple comparisons	Time F (11, 308)= 12.34, P<0.0001; Treatment F (1, 28)= 34.60, P<0.0001; Time x Treatment F (11, 308)= 6.393, P<0.0001
4	n	AM Body Weight	n= 11 CON-NDG^{Oxtr}, r	Mixed-effects analysis, Šídák's multiple comparisons	Time F (6.401, 108.3)= 8.097, P<0.0001; Treatment F (1, 17)= 22.16, P= 0.0002; Time x Treatment F (12, 203)= 2.887, P= 0.0011

Figure	Part		Sample size	Test	P-value, F- value, df, t-values
S1	b	LDB Light entries	n= 10 CON-NDG^{Oxtr}, n= 12 Gq-NDG^{Oxtr}	Unpaired two-tailed t-test	p= 0.0044, t= 3.206, df= 20
S1	c	LDB Light duration	n= 10 CON-NDG^{Oxtr}, n= 12 Gq-NDG^{Oxtr}	Unpaired two-tailed t-test	p= 0.0129, t= 2.408, df= 20
S1	e	EPM Open arm entries	n= 10 CON-NDG^{Oxtr}, n= 12 Gq-NDG^{Oxtr}	Unpaired two-tailed t-test	p= 0.0157, t= 2.314, df= 20
S1	f	EPM distance	n= 10 CON-NDG^{Oxtr}, n= 12 Gq-NDG^{Oxtr}	Unpaired two-tailed t-test	p= 0.0127, t= 2.737, df= 20
S1	h	OFA center entries	n= 14 CON-NDG^{Oxtr}, n= 13 Gq-NDG^{Oxtr}	Unpaired two-tailed t-test	p= 0.0022, t= 3.417, df= 25
S1	i	OFA distance	n= 14 CON-NDG^{Oxtr}, n= 13 Gq-NDG^{Oxtr}	Unpaired two-tailed t-test	p<0.0001, t= 5.788, df= 24

Figure	Part		Sample size	Test	P-value, F- value, df, t-values
S2	b	LDB Light entries	n= 8 CON-NDG^{Oxtr}, n= 7 Gq-NDG^{Oxtr}	Unpaired two-tailed t-test	p= 0.2422, t= 0.7198, df= 13
S2	c	LDB Light duration	n= 8 CON-NDG^{Oxtr}, n= 7 Gq-NDG^{Oxtr}	Unpaired two-tailed t-test	p= 0.8509, t= 0.1918, df= 13
S2	e	EPM Open arm entries	n= 8 CON-NDG^{Oxtr}, n= 7 Gq-NDG^{Oxtr}	Unpaired two-tailed t-test	p= 0.9142, t= 0.1099, df= 13
S2	f	EPM distance	n= 8 CON-NDG^{Oxtr}, n= 7 Gq-NDG^{Oxtr}	Unpaired two-tailed t-test	p= 0.2043, t= 1.337, df= 13
S2	h	Mouse preference	n= 8 CON-NDG^{Oxtr}, n= 7 Gq-NDG^{Oxtr}	Unpaired two-tailed t-test	p= 0.6850, t= 0.4149, df= 13
S2	i	Distance travelled	n= 8 CON-NDG^{Oxtr}, n= 7 Gq-NDG^{Oxtr}	Unpaired two-tailed t-test	p= 0.2905, t= 1.102, df= 13

Figure	Part		Sample size	Test	P-value, F- value, df, t-values
S3	a	PM Food Intake	n= 15 CON-NDG^{Oxtr}	Mixed-effects analysis, Dunnett's multiple comparisons	Time F (2.430, 102.1)= 625.2 , P<0.0001; Treatment F (2,42)= 0.6727, P= 0.5158; Time x Treatment F (42, 882)= 0.5109, P= 0.9960
S3	b	PM Temp	n= 7 CON-NDG^{Oxtr}	Mixed-effects analysis, Dunnett's multiple comparisons	Time F (4.551, 77.36)= 81.80 , P<0.0001; Treatment F (2,17)= 0.0.2157, P= 0.8081; Time x Treatment F (46, 391)= 1.633, P= 0.0076
S3	c	AM Food intake	n= 7 CON-NDG^{Oxtr}	Two-way RM ANOVA, Dunnett's multiple comparisons	Time F (1.347, 24.24)= 295.4, P<0.0001; Treatment F (2, 18)= 0.07495, P= 0.9281; Time x Treatment F (46,414)= 0.07942, P>0.9999
S3	d	AM Temp	n= 7 CON-NDG^{Oxtr}	Two-way RM ANOVA, Dunnett's multiple comparisons	Time F (4.984, 89.70)= 69.90, P<0.0001; Treatment F (2, 18)= 0.1290, P=0.8798; Time x Treatment F (46, 414)= 1.072, P= 0.6523
S3	e	PM VO_2	n= 15 CON-NDG^{Oxtr}	Mixed-effects analysis, Dunnett's multiple comparisons	Time F (6.349, 260.9)= 90.69, P<0.0001; Treatment F (2, 42)= 0.02596, P= 0.9744; Time x Treatment F (44, 904)= 0.6956, P= 0.9341
S3	f	PM RER	n= 15 CON-NDG^{Oxtr}	Mixed-effects analysis, Dunnett's multiple comparisons	Time F (4.271, 175.3)= 139.6, P<0.0001; Treatment F (2, 42)= 0.1282, P=0.8800; Time x Treatment F (44, 903)= 0.5772, P= 0.9879
S3	g	AM VO_2	n= 7 CON-NDG^{Oxtr}	Mixed-effects analysis, Dunnett's multiple comparisons	Time F (5.637, 101.0)= 66.56, P<0.0001; Treatment F (2, 18)= 0.03491, P= 0.9658; Time x Treatment F (46, 412)= 0.6729; P= 0.9503
S3	h	AM RER	n= 7 CON-NDG^{Oxtr}	Mixed-effects analysis, Dunnett's multiple comparisons	Time F (3.755, 67.27)= 108.2, P<0.0001; Treatment F (2, 18)= 0.4988, P= 0.6154; Time x Treatment F (46, 412)= 0.6736, P= 0.9499
S3	i	PM SBP	n= 6 CON-NDG^{Oxtr}	Two-way RM ANOVA, Dunnett's multiple comparisons	Time F (5.997, 89.90)= 38.09, P<0.0001; Treatment F (2, 15)= 0.05188, P= 0.9496; Time x Treatment F (46, 345)= 0.9880, P= 0.4990

Figure	Part		Sample size	Test	P-value, F- value, df, t-values
S3	j	PM HR	n= 7 CON-NDG^{Oxtr}	Two-way RM ANOVA, Dunnett's multiple comparisons	Time F (4.788, 86.18)= 29.45, P<0.0001; Treatment F (2, 18)= 0.02076, P= 0.9795; Time x Treatment F (46, 414)= 0.7147, P= 0.9193
S3	k	AM SBP	n= 6 CON-NDG^{Oxtr}	Two-way RM ANOVA, Dunnett's multiple comparisons	Time F (4.109, 61.63)= 30.60, P<0.0001; Treatment F (2, 15)= 0.3507, P= 0.7098; Time x Treatment (46, 345)= 1.233, P= 0.1527
S3	l	AM HR	n= 7 CON-NDG^{Oxtr}	Two-way RM ANOVA, Dunnett's multiple comparisons	Time F (6.154, 110.8)= 28.58, P<0.0001; Treatment F (2, 18)= 0.03459, P= 0.9661; Time x Treatment F (46, 414)= 1.073, P=0.3512
