# Supplementary material for: Mechanosensation of the heart and gut elicits hypometabolism and vigilance in mice
Source: bioRxiv. 2023 Jul 1:2023.06.29.547073. Preprint. [Version 1] doi: 10.1101/2023.06.29.547073 (PMC10327188; doi:10.1101/2023.06.29.547073)
Supplement: Supplement 2 [file NIHPP2023.06.29.547073v1-supplement-2.pdf]

bioRxiv preprint doi: https://doi.org/10.1101/2023.06.29.547073; this version posted July 1, 2023. The copyright holder for this preprint (which was not certified by peer review) is the author/funder, who has granted bioRxiv a license to display the preprint in perpetuity. It is made available under aCC-BY 4.0 International license.

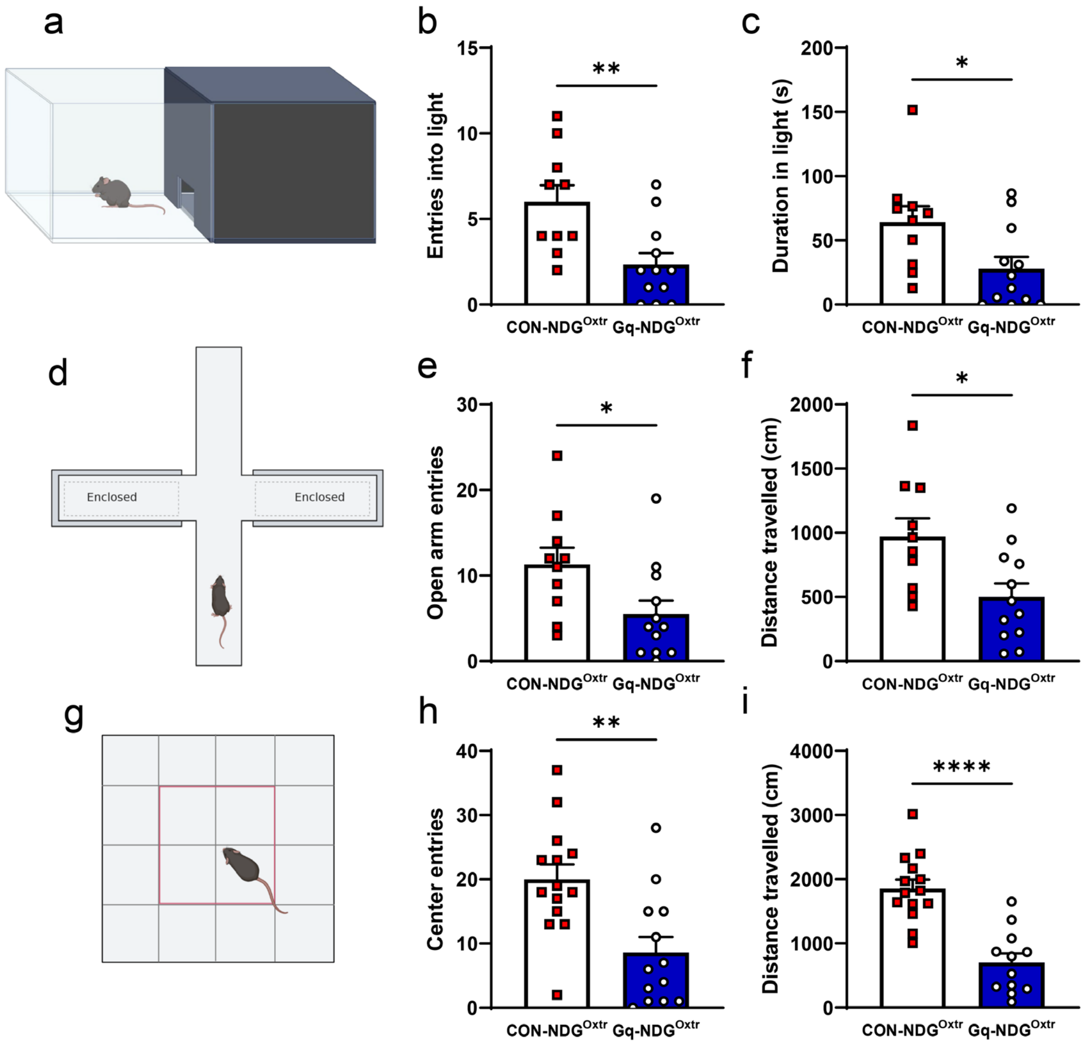

__Supplemental Figure 1__. **Acute activation of NDG^{Oxtr} induces anxiety-like behavior and reduces locomotor activity. a,d,g** are schematics of the light dark box (LDB), elevated plus maze (EPM) and open field arena (OFA), respectively. Gq-NDG^{Oxtr} made fewer entries into the light **(b)** and spent less time in the light side (c) of the LDB. Gq-NDG^{Oxtr} mice made fewer entries **(e)** and travelled less in the EPM **(f)**. Gq-NDG^{Oxtr} made fewer center entries (g) and travelled less in the OFA (h). Data shown as mean ± s.e.m., n= 10-14 CON-NDG^{Oxtr}, 12-13 Gq-NDG^{Oxtr}. Unpaired one-tailed t-tests. *$p<0.05$, **$p<0.01$, ****$p<0.0001$. Schematics (a,d,g) made with Biorender.com.

bioRxiv preprint doi: https://doi.org/10.1101/2023.06.29.547073; this version posted July 1, 2023. The copyright holder for this preprint (which was not certified by peer review) is the author/funder, who has granted bioRxiv a license to display the preprint in perpetuity. It is made available under aCC-BY 4.0 International license.

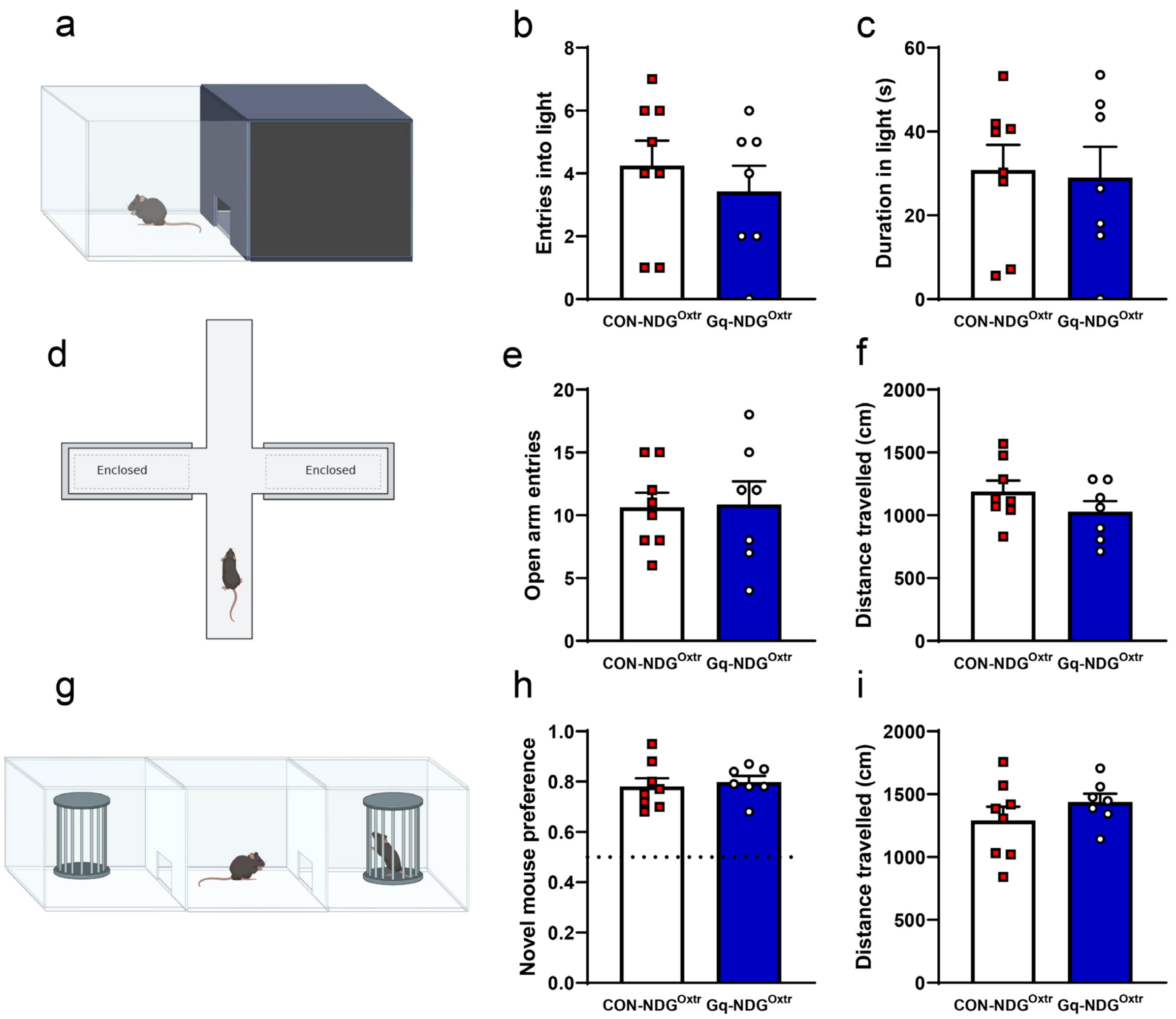

<u>Supplemental Figure 2</u>. Chronic activation of NDG^{Oxtr} does not induce anxiety-like behavior or reduce social preference. a,d,g are schematics of the light dark box (LDB), elevated plus maze (EPM) and social interaction chamber, respectively. Entries into (b) and durations of time (c) spent in the light side of the LDB did not differ between groups. Open arm entries (e) and distance travelled (f)in the EPM did not differ between groups. Chronic activation of NDG^{Oxtr} did not affect preference investigating a novel mouse (h) or distanced travelled (i) in the 3-chamber social interaction test. Data shown as mean ± s.e.m., n= 8 CON-NDG^{Oxtr}, 7 Gq-NDG^{Oxtr}. Unpaired, one-tailed t-tests. Schematics (a,d,g) made with Biorender.com.

bioRxiv preprint doi: https://doi.org/10.1101/2023.06.29.547073; this version posted July 1, 2023. The copyright holder for this preprint (which was not certified by peer review) is the author/funder, who has granted bioRxiv a license to display the preprint in perpetuity. It is made available under aCC-BY 4.0 International license.

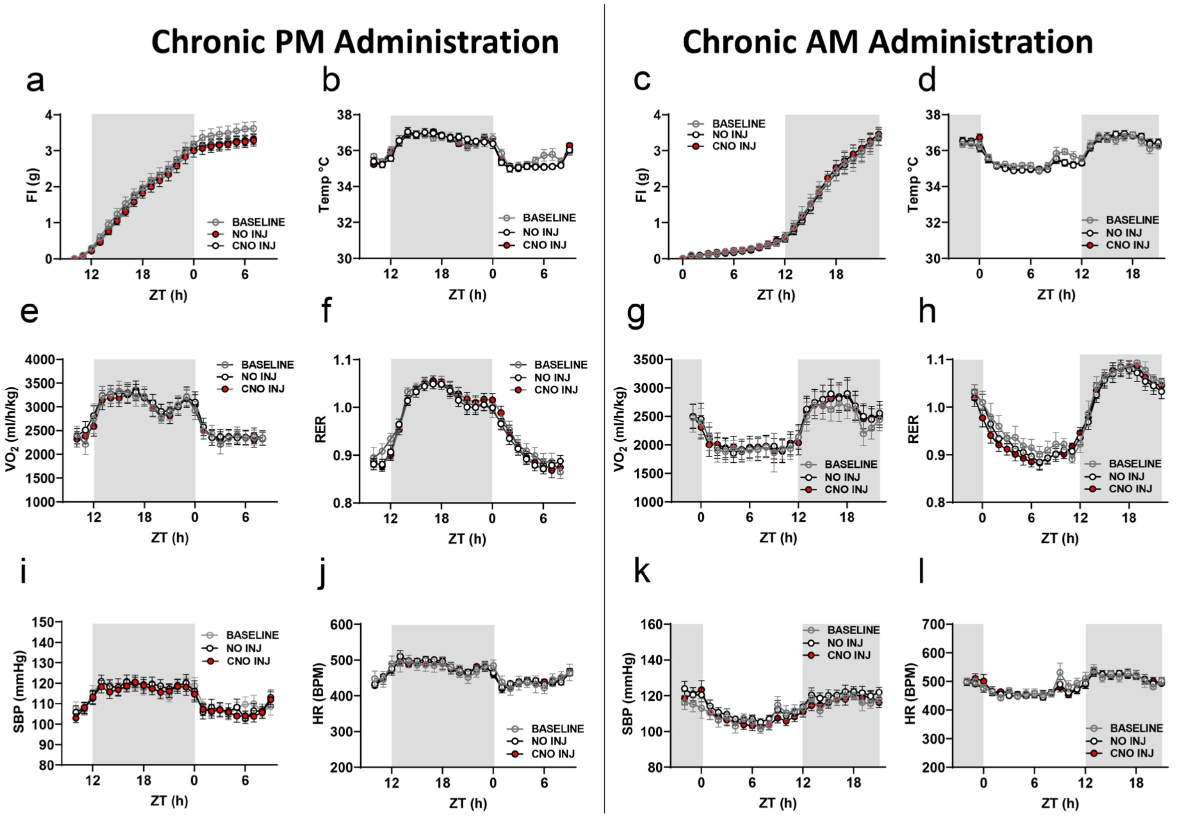

<u>Supplemental Figure 3</u>. Chronic administration of CNO (0.3 mg/kg, i.p.) to CON-NDG^{Oxtr} (lacking Gq DREADDs) has no effect on food intake (a,c), core body temperature (b,d), oxygen consumption (e,g) or RER (f,h), SBP (i,k) or HR (j,l) when given 1h prior to onset of dark phase (left panel) or light phase (right panel). CON-NDG^{Oxtr} data are averaged between baseline (2 days), no injection (5 days) and CNO injection days (6 days). All figures express mean ± s.e.m. n= 15 CON (a,e,f), 7 CON (b,c,d,g,h,I,j,k,l)
